# Toward the realization of cardiac regenerative medicine using pluripotent stem cells

**DOI:** 10.1186/s41232-019-0110-4

**Published:** 2020-01-13

**Authors:** Yoshikazu Kishino, Jun Fujita, Shugo Tohyama, Marina Okada, Sho Tanosaki, Shota Someya, Keiichi Fukuda

**Affiliations:** Department of Cardiology, Keio University School of Medicine, 35 Shinanomachi, Shinjuku, Tokyo, 160-8582 Japan

**Keywords:** Regenerative medicine, Induced pluripotent stem cell, Cardiomyocyte

## Abstract

Heart transplantation (HT) is the only radical treatment available for patients with end-stage heart failure that is refractory to optimal medical treatment and device therapies. However, HT as a therapeutic option is limited by marked donor shortage. To overcome this difficulty, regenerative medicine using human-induced pluripotent stem cells (hiPSCs) has drawn increasing attention as an alternative to HT.

Several issues including the preparation of clinical-grade hiPSCs, methods for large-scale culture and production of hiPSCs and cardiomyocytes, prevention of tumorigenesis secondary to contamination of undifferentiated stem cells and non-cardiomyocytes, and establishment of an effective transplantation strategy need to be addressed to fulfill this unmet medical need. The ongoing rapid technological advances in hiPSC research have been directed toward the clinical application of this technology, and currently, most issues have been satisfactorily addressed. Cell therapy using hiPSC-derived cardiomyocytes is expected to serve as an integral component of realistic medicine in the near future and is being potentially viewed as a treatment that would revolutionize the management of patients with severe heart failure.

## Background

In 2006, Yamanaka et al. introduced the genes *Oct3/4*, *Sox2*, *Klf4*, and *c-Myc* (referred to as Yamanaka factors) into somatic cells in mice and successfully developed induced pluripotent stem cells (iPSCs) [1], which showed properties similar to those of embryonic stem cells (ESCs). In 2007, they developed human-iPSCs (hiPSCs) [2]. It is possible to elucidate the pathophysiology of several unknown genetic diseases using patient-derived hiPSCs, and these are also useful for novel drug screening. Thus, the emergence of hiPSCs is a promising therapeutic approach in patients with diseases that were previously considered incurable. Evaluation of the responsiveness of patient-derived hiPSCs to drugs can determine the role of these cells in personalized medicine. Moreover, hiPSCs are drawing increasing attention as a revolutionary approach toward the rapid realization of regenerative medicine. This unique technology overcomes the challenges affecting regenerative medicine research such as ethical issues and immune rejection reactions, which serve as significant drawbacks of ESCs derived from the inner cell mass that forms a part of the embryo (blastocyst stage).

End-stage heart failure is a significant contributor to the cardiovascular disease burden in adults. Unfortunately, this condition is refractory to medical treatment and device therapies. Heart transplantation (HT) is the only radical treatment available in the present era. However, a marked shortage of donor hearts limits the availability of HT as a therapeutic option, particularly in Japan. Currently, the number of patients undergoing HT is < 100, and the waiting period to register for transplantation is > 3 years (The Registry Report of Heart Transplantation in Japan 2016). Given this scenario, hiPSC-derived cardiomyocytes are considered an ideal cell source in patients requiring HT for severe heart failure [3].

**Fig. 1:**
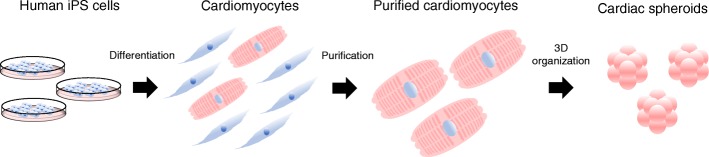
Strategy of cardiac regenerative therapy using human iPSC-derived cardiomyocytes. iPSC, induced pluripotent stem cell

In this review, we have discussed the current scenario with regard to the utility of hiPSC-derived cardiomyocytes in cardiac regenerative medicine, as well as their clinical application (Fig. 1).

## Main text

### Protocols for cardiac differentiation of human pluripotent stem cells

Several researchers have reported cardiac differentiation of pluripotent stem cells (PSCs) to artificially generate human cardiomyocytes (Table 1). Regarding the induction of cardiomyocytes from human-PSCs (hPSCs), these can be induced to differentiate into cardiomyocytes at different sites within the heart, such as the atria, ventricles, and other such structures. Reportedly, these cells show the same characteristic electrical activity as demonstrated by human cardiomyocytes [19]. Protocols for the differentiation of hiPSCs into cardiomyocytes have been established based on the development and differentiation of the heart [18]. Currently, three- and two-dimensional culture methods are available for cardiac differentiation. The three-dimensional culture method generates large quantities of cardiomyocytes by suspension culture using a bioreactor or spinner flask [20]. However, this technology is expensive because it requires the use of recombinant proteins, such as bone morphogenetic proteins (which belong to the transforming growth factor-β superfamily), to induce differentiation into the mesoderm. In contrast, 2-dimensional culture involves differentiation methods that use low-molecular-weight compounds such as CHIR99021 (an inhibitor of glycogen synthase kinase 3β) and inhibitors of Wnt, such as IWR-1 and IWP-2. This technology is a cost-effective option for differentiation into cardiomyocytes. Furthermore, two-dimensional culture using multilayer culture plates with active gas ventilation has enabled the generation of large quantities of cardiomyocytes that are required for transplantation [17]. No method can achieve 100% efficiency in cardiomyocyte differentiation, and variations are observed among hiPSC lines and passage numbers; therefore, optimization of the cell differentiation protocol is necessary using specific quantities of pre-optimized reagents to support differentiation into the desired cell types. Development of an efficient method to trigger cardiac differentiation is essential for the large-scale mechanized production of these cells for the realization of transplantation therapy using hiPSC-derived cardiomyocytes. Further research is warranted to develop simpler and more efficient and stable methods.

**Table 1:** Cardiac differentiation protocols

Publication	Differentiation method	Media	Coating matrix	Mesoderm induction	Cardiac specification	Differentiation efficiency
Kehat et al. [4]	EB formation	KO DMEM + 20%FBS	Gelatin	NA	NA	8.1% beating EBs
Laflamme et al. [5]	Monolayer	RPMI1640 + B27	Matrigel	Activin A, BMP4	NA	> 30%β-MHC(+)CMs
Yang et al. [6]	EB formation	Stempro34	Gelatin	Activin A, BMP4, bFGF	VEGF, DKK1, bFGF	40–50% TNNT(+)CMs
Tran et al. [7]	EB formation	KO DMEM + 15%FBS	Gelatin	WNT3A	NA	EB formation 10-folds increase (vs control)
Elliot et al. [8]	EB formation or monolayer	LI-APEL	Matrigel	Activin A, BMP4, bFGF, VEGF, SCF, WNT3A	NA	EB:38% Nkx2.5(+) cells Monolayer:24% Nkx2.5(+) cells
Kattman et al. [9]	EB formation	Stempro34	NA	Activin A, BMP4, bFGF	VEGF, DKK1, TGFβi, BMPi	> 60% TNNT(+)CMs
Zhang et al. [10]	Monolayer	RPMI1640 + B27	Gelatin	Activin A, BMP4, bFGF	NOGGIN, RA/RAi, DKK1	RA:50.7% ± 1.7% RAi:64.7% ± 0.9%
Willems et al. [11]	EB formation	Stempro34	Gelatin	Activin A, BMP4, bFGF	bFGF, IWR-1, triiodothyronine	30%MYH6(+)CMs
Zhang et al. [12]	Monolayer (matrix sandwich)	RPMI1640 + B27 insulin(−)	Matrigel	Activin A, BMP4, bFGF	NA	40–92% TNNT(+)CMs
Lian et al. [13]	Monolayer	RPMI1640B27 insulin(−)	Matrigel or Synthemax	CHIR99021	IWP2	85%TNNT(+)CMs
Burridge et al. [14]	Monolayer	CDM3	Vitronectin	CHIR99021	Wnt-C59	80–95% TNNT(+)CMs
Devalla et al. [15]	EB formation	BPEL	Gelatin	Activin A, BMP4, CHIR99021, SCF, VEGF	RA	50%Nkx2.5(+)cells
Protze et al. [16]	EB formation	Stempro34	NA	Activin A, BMP4, bFGF	IWP2, VEGF, BMP4, RA, bFGFi, TGFβi	5%Nkx2.5(+)cells 55%Nkx2.5(−)SANLPCs
Tohyama et al. [17]	Monolayer (multilayer plates)	RPMI1640 + B27 insulin(−)	Fibronectin or type1 collagen	CHIR99021, BMP4	IWR-1	80%TNNT(+)CMs

Modified table taken from [18]

Abbreviations: *bFGF* basic fibroblast growth factor, *bFGFi* bFGF inhibitor, *BMP* bone morphologic protein, *BMPi* BMP inhibitor, *CMs* cardiomyocytes, *DKK* Dickkopf, *DMSO* dimethyl sulfoxide, *EB* embryoid body, *FBS* fetal bovine serum, *KO* knockout, *MLC* myosin light chain, *N/A* not applicable or not available, *TGFβ* transforming growth factor β, *TGFβi* TGFβ inhibitor, *TNNT* troponin T, *SALPC* sinoatrial node-like progenitor cells, *SCF* stem cell factor, *VEGF* vascular endothelial growth factor

### Cardiomyocyte purification system

The currently available cardiomyocyte differentiation methods are highly efficient in producing cardiomyocytes. However, if all cells do not differentiate into cardiomyocytes (if the rate of differentiation is not 100%), there exists an increased risk of tumorigenesis secondary to contamination with non-cardiomyocytes and undifferentiated cells at the time of transplantation. Unfortunately, this factor is a significant barrier to the realization of cardiac regenerative medicine. Therefore, safe transplantation without risk of tumorigenesis necessitates the removal of undifferentiated stem cells and non-cardiomyocytes. Various methods have been reported for the removal of undifferentiated stem cells [21–23] to prevent teratoma formation. Among these studies, we identified glypican-3 (GPC3), a known carcinoembryonic antigen, as a pluripotent state-specific immunogenic antigen. Moreover, we also confirmed that GPC3-reactive cytotoxic T lymphocytes (CTLs) selectively removed undifferentiated PSCs from hiPSC-derivatives in vitro and inhibited tumor formation in vivo [24]. However, contaminating non-cardiomyocytes undergoing differentiation may cause tumorigenesis of non-cardiomyocytes. Therefore, we evaluated the applicability of a method for purification of cardiomyocytes alone as a safer transplantation method. Thus, we created a metabolic environment that was conducive to the survival of cardiomyocytes but not undifferentiated stem cells and non-cardiomyocytes. We developed a cardiomyocyte purification medium containing glucose-free lactic acid, which enabled purification of only cardiomyocytes based on the difference in metabolism [25]. Following intensive research focusing on amino acid metabolism, it is known that glutamine is essential for the survival of hiPSCs and that the use of glucose and glutamine-free lactic acid-supplemented medium improves the efficiency of the cardiomyocyte purification method. This knowledge was useful in removing undifferentiated stem cells more efficiently for the purification of cardiomyocytes in a clinical setting [26]. Thus, only cardiomyocytes can be selected in large quantities in a cost-effective manner without using genetic modification technology or fluorescence-activated cell sorting. Moreover, tumor formation (teratomas) was not observed even after transplantation of these cells into immunodeficient mice. Therefore, it is reasonable to conclude that this metabolic selection method for differentiated cardiomyocytes can ensure safe regenerative cardiomyocyte transplantation.

### Strategy for the transplantation of induced pluripotent stem cell-derived cardiomyocytes

A previous study investigating regenerative therapy with cardiomyocyte transplantation has reported that iPSC-derived cardiomyocytes prepared from skin fibroblasts in patients with heart failure were transplanted into rat hearts and were successfully engrafted [27]. Another study showed that human-ESC (hESC)-derived myocardium transplanted in a guinea pig myocardial infarction model led to improved cardiac function and decrease of ventricular arrhythmias after transplantation [28]. These results indicate the potential utility and feasibility of ESC or iPSC-derived cardiomyocyte transplantation therapy for myocardial regeneration (Table 2). To date, heart cell transplantation is performed by direct injection of the cell suspension into the heart via a syringe, although there is room for improvement in the cell survival rate. Most transplanted cells were observed to be necrotic or an efflux [40]. This observation can be attributed to the fact that the transplanted cardiomyocytes flow out of the myocardium secondary to the beating of the heart, resulting in a low-survival rate. Notably, cell sheet technology is a method of transplantation that involves stacking of multilayered cardiomyocyte sheets to form a scaffold that is transplanted onto the epicardium [41]. However, with this method, the transplanted myocardium may not be electrophysiologically synchronized with the recipient’s heart because the epicardium is an electrically insulating tissue. We produced cardiac spheroids with purified cardiomyocytes (approximately 200 μm in diameter) and observed that transplantation of these significantly improved the engraftment rate [29, 42]. Cardiomyocyte aggregates do not flow out owing to increased cell adhesion and secretion of cell growth factor and increased cell mass size. Additionally, a recent study investigating the effectiveness of hiPSC-derived cardiomyocyte transplantation into a large animal myocardial infarction model such as a pig and monkey model has been reported as a preclinical study to evaluate the safety and efficacy of the clinical application of this approach [30–33], and it is expected that human studies would soon be reported.

**Table 2:** Transplantation protocols

Publication	Transplantation Method	Recipient Animal Species	Recipient Model	Donor Cell Type	Number of Donor Cells	Transplantation Efficacy
Hattori F et al. [29]	Direct cell injection	Mouse	Healthy heart	mESC-CMs hESC-CMs	NA	NA
Zwi-Dantsis L et al. [27]	Direct cell injection	Rat	Healthy heart	hiPSC-CMs	1.5×10^5^	Develop gap junctions between donor hiPSC-CMs and host rat CMs
Shiba et al. [28]	Direct cell injection	Pig	Cryoinjury induced infarcted heart	hESC-CMs	1×10^8^	Reduce ventricular tachycardia Improve left ventricle fractional shortening
Ye et al. [30]	Direct cell injection	Pig	LAD ligation induced infarcted heart	hiPSC-CMs hiPSC-EC hiPSC-SMC fibrin patch	2×10^6^ 2×10^6^ 2×10^6^	Improve left ventricle ejection fraction and infarct size
Chong JJ et al. [31]	Direct cell injection	Monkey	LAD balloon occlusion induced infarcted heart	hESC-CMs	NA	Electromechanical coupling between graft and host myocytes
Shiba et al. [32]	Direct cell injection	Monkey	LAD ligation induced infarcted heart	hiPSC-CMs	4×10^8^	Increase ventricular tachycardia transiently Improve left ventricle ejection fraction and fractional shortening
Liu YW et al. [33]	Direct cell injection	Monkey	LAD balloon occlusion induced infarcted heart	hESC-CMs	7.5×10^8^	Increase ventricular tachycardia transiently Improve left ventricle ejection fraction
Tabei et al. [42]	Direct cell injection	Pig	Healthy heart	hiPSC-CM spheroids	1×10^7^	The combination of the newly developed transplant device and spheroid formation promotes the retention of transplanted CMs
Kimura et al. [34]	Pericardial endoscopy and direct injection	Pig	Healthy heart	NA	NA	NA
Masumoto et al. [35]	Cell sheets	Rat	LAD ligation induced infarcted heart	mESC-CMs mESC-ECs&MCs	5×10^5^ 5×10^5^	Improve left ventricle systolic function and infarct size
Suzuki et al. [36]	Cell sheets Omentopexy	Rat	LAD ligation induced infarcted heart	Neonatal CMs	5.6×10^5^/cm^2^	Improve left ventricle ejection fraction
Kashiyama et al. [37]	Cell sheets	Monkey	LAD ligation induced infarcted heart	mkiPSC-CMs	3.6×10^6^/sheet	Improve left ventricle systolic function
Ott HC et al. [38]	Cell scaffolding Recellularization	Rat	Decellularized heart	Neonatal CMs Fibrocytes ECs SMCs	5-7.5×10^7^	Generate working recellularized heart
Lu et al. [39]	Cell scaffolding Recellularization	Mouse	Decellularized heart	hiPSC-MCPs	1×10^7^	Generate working recellularized heart having responsiveness to drugs

*Abbreviations*: *hiPSC* human induced pluripotent stem cell, *mESC* mouse embryonic stem cell, *hESC* human embryonic stem cell, *mkiPSC* monkey induced pluripotent stem cell, *CMs* cardiomyocytes, *ECs* endothelial cells, *SMCs* smooth muscle cells, *MCs* vascular mural cells, *MCPs* multipotential cardiovascular progenitors, *LAD* left anterior descending artery, *N/A* not applicable or not available

### Adverse effects of cell transplantation

**Fig. 2:**
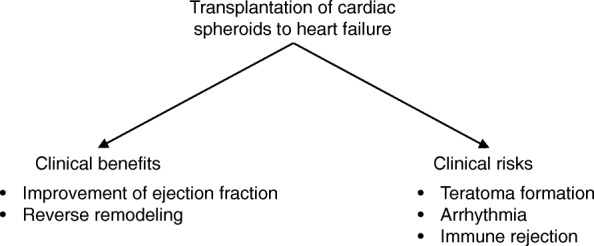
Clinical benefits and risks of cardiac regenerative therapy

Arrhythmias and immune response-mediated transplant rejection are serious adverse events associated with cardiomyocyte transplantation (Fig. 2). A previous study has reported the development of arrhythmias in patients with severe heart failure who received an injection of skeletal myoblasts [43]. Connexin 43 and N-cadherin, which are essential to establish electrophysiological connections between cardiomyocytes, are not expressed in myoblasts, and automatism associated with myoblasts can precipitate arrhythmias [44]. Previous studies have shown that cardiomyocytes can establish connections with the host heart, and hESC-derived cardiomyocytes can establish electrical connections with neonatal rat cardiomyocytes and become synchronized to beat in vitro [45]. Additionally, it has been shown that transplanted cardiomyocytes demonstrated electrical coupling with the host heart after hESC-derived cardiomyocytes were engrafted to guinea pig hearts [28]. However, several studies have also reported the development of ventricular arrhythmia within the first 2 weeks to 1 month after transplantation into a recipient’s heart, which however disappeared a month after cell transplantation [31, 46]. These data suggest that the arrhythmogenicity of hPSC-derived cardiomyocytes in vivo remains controversial and that it is necessary to closely monitor the heart for arrhythmias after cell transplantation in humans.

Autologous transplantation of iPSCs can realize the goal of cell transplantation without the institution of immunosuppressive therapy. In cases of allogeneic transplantation of iPSCs, controlling the immune response in a recipient is essential for successful engraftment of transplanted cardiomyocytes. Maintaining an iPSC bank is a useful clinical strategy to obtain human leukocyte antigen (HLA)-matched iPSCs, which will obviate the need for immunosuppressant administration in the recipient [47]. However, it should be noted that non-HLA-matched allogeneic cell transplantation requires the administration of a complete immunosuppressive regimen. The immunosuppressive regimen is essentially the same as that prescribed in patients undergoing HT, because the optimal immunosuppressive regimen for cardiac cell transplantation remains unknown. Notably, immunosuppressive therapies can cause adverse effects, such as severe infection and malignancy.

## Conclusions

The realization of cardiac regenerative medicine using hiPSCs requires the efficient and cost-effective large-scale production of cardiomyocytes. Avoiding contamination with residual undifferentiated stem cells and non-cardiomyocytes is essential and this is the biggest safety challenge in this field. Fortunately, technological advances have facilitated effective strategies for the resolution of these issues. Several preclinical studies performed in large animals (guinea pig and monkey) are ongoing, and much progress has been reported in this realm. It is expected that following complete verification of safety and efficacy, cardiac regenerative medicine using hiPSCs will show wide clinical applicability in humans.

## Data Availability

Not applicable.

## Abbreviations

BMP: Bone morphogenetic proteins
ESC: Embryonic stem cell
GPC3: Glypican-3
iPSC: Induced pluripotent stem cell
PSC: Pluripotent stem cell
